# Adherence to psychiatric medications and diagnosis

**DOI:** 10.1192/j.eurpsy.2023.554

**Published:** 2023-07-19

**Authors:** C. González Navarro, A. Bilbao Idarraga, I. Alonso Salas, L. Morado San segundo, A. López Fariña, U. López Puentes, B. Samsó Martínez, R. F. Lopez Brokate, T. Ruiz de Azua Aspizua, E. M. Garnica de Cos, U. Ortega Pozas

**Affiliations:** RED DE SALUD MENTAL DE BIZKAIA, ZAMUDIO, Spain

## Abstract

**Introduction:**

Patients with mental disorders frequently become non-adherent during their long term prescribed treatment. This situation frequently triggers clinical worsening and hospital admission. Therefore, non-adherence may result in poorer long term clinical outcomes and has economic implications for health-care providers (Carlos De las Cuevas et al. Neuropsychopharmacol Hung 2021; 23(4):347-362).

**Objectives:**

- To describe the adherence to oral and long acting injectable treatment in the sample of patients that were admitted to the short stay hospital unit during the period of study.

- To describe the adherence to treatment amongst psychiatric diagnosis in the sample of study.

**Methods:**

It was a retrospective observational study with a duration of three months. Data was collected from all patients admitted to the short stay hospital unit during the period of study and there were no specific exclusion criteria. Descriptive statistics were performed. To assess the adherence to pharmachological treatment the patient report, the family report and the pharmacy dispensation according to the existent informatic prescription platform was considered. Regarding the long acting injectable treatment the formulary of administration in the clinical history was checked.

**Results:**

During the period of study 172 patients were admitted to the short stay hospital unit. Of those, 146 patients had a previous pharmacologic prescription. Data of treatment was not possible to obtain in 7 patients. In the sample of study, 83.5% were on oral and 16.5% on long acting injectable treatment. The general adherence to treatment in the sample was 61.87%. In the oral treatment group the adherence was 58.4% and in the long acting injectable treatment group was 65.2%.

Amongst the different psychiatric diagnoses the outcomes of adherence to treatment were: 60.4% in schizophrenia and related psychosis, 62.5% in bipolar disorder, 78.6% in depression, 58.3% in personality disorders and 62% in addictive disorders.

**Conclusions:**

In our descriptive study adherence to treatment was higher in the long acting injectable treatment group, agreeing with the existent scientific literature.

The results of adherence for schizophrenia and bipolar disorder are similar to the ones found in scientific literature but differ from the ones for depression, being higher in our sample (Judit Lazary et al. Neuropsychopharmacol Hung 2021;23(4): 347-362). Moreover, in scientific literature it is found a similar prevalence of adherence across diagnosis (for schizophrenia, bipolar disorder and depression) whereas in our sample patients with depression showed a different and higher adherence to treatment (Judit Lazary et al. Neuropsychopharmacol Hung 2021;23(4): 347-362). In our sample, patients with personality disorders had the lowest adherence to treatment.

**Disclosure of Interest:**

None Declared

